# Science and the Nation

**Published:** 1920-06-19

**Authors:** 


### SCIENCE AND THE NATION.

At the recent meeting of the British Science Guild, Lord Sydenham opened by quoting the remark of a distinguished American friend of this country: "To me, the greatest problem of the hour is the strike, which I think a greater menace to the world than Kaiserism ever was, and the fundamental cause of the strike evil is the perverted psychology of the working classes, which in turn is the inevitable result of conditions brought about by the substitution of the machine for the man, from which no escape seems possible." The strike evil has grown to dangerous dimensions in this country; from being the legitimate weapon of the weak against injustice which could not be otherwise redressed, it has become the instrument of caprice or blind passion, without waiting for facts or to seek conciliation. Even the advice of the men's leaders may be set at naught by the workers. Direct action is a flagrant violation of the principles of democracy and is economically disastrous. It is impossible to estimate the total national loss caused by strikes since the Armistice. When foreign competition again begins to be felt, strikes or industrial conflicts will gravely injure our valuable export trade. The present economic conditions can only be conquered by honest work of hand and brain, by economy and thrift on the part of all classes. While the newly enriched people are spending lavishly on superfluities, we see the bricklayer restricting his efforts to one-third, despite the need of houses for his fellow-workers, and the coal-miner, despite increasing numbers, shorter hours, and good wages, does not approach his pre-war output. The menace to civilisation arises from a perverted mentality affecting all classes. The reign of the machine has changed the whole life of the country, and it becomes increasingly imperious; it has transferred power to the towns, leaving a relatively small rural population, with the relegation of agriculture to a subordinate place. In the rapid growth of towns which followed poor provision was made for sanitary, in many cases even decent, conditions of living; and from this neglect a host of evils sprang, one of which was our large dependence on imported food, paid for by our exports, and ensured by our naval supremacy. The race for wealth causes, in too many cases, forgetfulness of the responsibilities involved. The land worker cannot fail to understand the increase of production which would follow the scientific application of capital to industry; but the land worker is now only a small proportion of the total, and the psychology of the labourer is mostly that of the town worker. In former times the employer often worked with his men, but he is now largely replaced by a Board of Directors which sits in secret, and workers not familiar with brain activities are confused by the change. The perverted mentality may have affected both capital and labour. Only by goodwill and a readiness for mutual concession can the spirit of unreasoning hostility be exorcised.

The question arises, Can science help to provide the antidote? While science is transforming the national life, there is no corresponding development of science training in Governmental and administrative circles; and even to-day, when science is so all-important, a mere moderate knowledge of the principles of science is not considered a necessary part of the equipment for Governmental and ambassadorial service. Even the Board of Education is swamped by officials whose training was literary only, and who had inherited a traditional disregard of science as an educative force. It was deliberately decided that cotton was not required for the manufacture of nitro-cellulose and might safely be admitted to Germany; also that lard, as it could not be used in making nitro-glycerine, need not be regarded as contraband! Examples of grave decisions having been taken without adequate knowledge and in defiance of the scientific spirit can be readily multiplied.

The war has resulted by now in the calling in of science experts. Sir Joseph Thomson wrote that on the General Staff at the War Office there was no officer with any real knowledge of science or appreciation of scientific questions, and in contrast it is good to see the new impetus which research has received, but there must be the application of the scientific spirit to the solution of national problems, which is the object of this Guild.

This will only be attained by radical changes in our system of education, and there must be trained specialists wielding adequate authority in the national councils. The scientific habit of thought is acquired only by the recognition of eternal and inexorable laws, by the pursuit of truth for truth's sake, by insistence on proved facts, and distrust of mere phrases. Never was there such a time to "prove all things and hold fast to that which is good." Goethe has well said "There is no more dreadful sight than ignorance in action."

